# Beyond Human Papillomavirus (HPV): Detection of EBV and Polyomaviruses in Cervical and Anal Samples

**DOI:** 10.1002/jmv.70909

**Published:** 2026-04-16

**Authors:** Ana Carolina Silva Guimarães, Luana L. S. Rodrigues, Letícia Bomfim Campos, Katrini G. Martinelli, Nathália Silva Carlos Oliveira, W. Martin Kast, Charlotte A. Gaydos, Carlos Silva de Jesus, José H. Pilotto, Mariza G. Morgado, Tarik Gheit, Vanessa Salete de Paula

**Affiliations:** ^1^ Molecular Virology and Parasitology Laboratory Oswaldo Cruz Institute Rio de Janeiro Brazil; ^2^ Clinical Analysis Laboratory—Institute of Collective Health Federal University of Western Pará Pará Brazil; ^3^ Graduate Program in Health Sciences—Institute of Collective Health Federal University of Western Pará Para Brazil; ^4^ Laboratory of AIDS and Molecular Immunology Oswaldo Cruz Institute Rio de Janeiro Brazil; ^5^ Department of Collective Health Federal University of Espírito Santo Santo Brazil; ^6^ Department of Pathology—School of Medicine Fluminense Federal University Rio de Janeiro Brazil; ^7^ Department of Obstetrics and Gynecology University of Southern California Los Angeles USA; ^8^ Norris Comprehensive Cancer Center University of Southern California Los Angeles USA; ^9^ Department of Molecular Microbiology and Immunology University of Southern California Los Angeles USA; ^10^ Division of Infectious Diseases Johns Hopkins University, School of Medicine Baltimore USA; ^11^ International Agency for Research on Cancer—IARC Lyon France

**Keywords:** Amazon, EBV, HPV, polyomaviruses, women

## Abstract

Colorectal and cervical cancers represent an important public health problem worldwide, particularly in Brazil. Colorectal cancer is one of the most frequently diagnosed malignancies worldwide and the most common cancer in both men and woman in Brazil. Although cervical cancer also remains a significant public health concern in the female population due to its high incidence and mortality rates. Epstein‐Barr virus (EBV), and Polyomaviruses (JCPyV and BKPyV) are considered as oncoviruses with a prevalence of 90% in the adult population. Studies suggest that coinfection with Human papillomavirus (HPV) and EBV, JCPyV and BKPyV can be associated with anal cancer, with HPV being the dominant viral cause. Although in cervical cancer, this coinfection is often observed, and it may enhance cervical oncogenic potential. These facts are particularly concerning in women living with HIV, who are more susceptible to persistent HPV infections and HPV‐related cancers. This study aimed to investigate EBV, JCPyV and BKPyV presence in HPV‐infected women living with HIV. This cross‐sectional study collected 33 cervical and 30 anal scrapings samples, as well as 26 paired samples from both sites from HPV‐positive women living with HIV. Viral detection and quantification for EBV, JCPyV and BKPyV were performed using real‐time polymerase chain reaction (qPCR), while HPV detection and genotyping utilized the Novaplex^TM^ assay. EBV was detected in 33.3% of anal scraping and 21.2% in cervical scraping. HPV 51 and HPV 56 were most frequently detected in anal and cervical scrapings, respectively. The EBV mean viral load was higher in cervical scraping (2.27 × 10^5 copies/mL) when compared to anal scraping (1.19 × 10^5 copies/mL) in relation of JCPyV and BKPyV viral loads. Regarding coinfections, it was found that EBV presented a high coinfection rate with high‐risk HPV genotypes (hrHPV) (14.6%) with HPV 31 in both samples. This study provided valuable data about the frequency of EBV in anal and cervical HPV‐positive women. The elevated viral load of EBV in cervical scrapings may suggest a potential role in modulating the local microenvironment and warrants further investigation into the synergistic effects of EBV and HPV in anogenital and cervical carcinogenesis. Overall, the results of this study emphasize the need for further investigation into the role of these viruses in the progression of cervical lesions.

## 1 Introduction

Colorectal and cervical cancers represent a significant public health burden worldwide due to their high incidence and mortality rates. Colorectal cancer is the third‐most common cancer diagnosed worldwide and the second most common cause of cancer‐related death, accounting for approximately 10% of all cancer cases according to National Cancer Institute (NIH), International Agency of Research on Cancer (IARC) and World Health Organization (WHO) [1, 2, 3]. According to the Global Cancer Observatory (GLOBOCAN), epidemiological data accounts for over 1.9 million new cases and 935,000 deaths worldwide [2, 4]. In Brazil, colorectal cancer is the second most common cancer among men and women, excluding non‐melanoma skin cancers [4]. It is estimated ~45,000 new cases annually in Brazil, of which 23,660 occur in women, with higher incidence and mortality rates in the South and Southeast [3, 4]. Although cervical cancer also represents a major oncological concern in the female population and is recognized as a significant public health problem due to its elevated incidence and mortality rates [5]. In Brazil, it is estimated that approximately 17,010 new cases occurred between 2023 and 2025, leading to around 6,606 deaths, with the highest rates in the Northern region, corresponding to the Amazon [5, 6]. Some risk factors like coinfections with HIV, immune system suppression, smoking, oral contraceptives, and many pregnancies throughout life and oncoviruses can lead to the development of cervical cancer [7, 8].

Among these facts, it is estimated that 12% of human cancers are caused by oncoviruses infection with more than 80% of cases occurring in the developing world [9]. The study about oncoviruses is limited to animal models, the disparate nature of virus‐induced cancers, the very distinct types of viruses associated with them and the complexity of the process of the virus‐host cell interactions leading to cancer development [9]. Oncoviruses such as Epstein‐barr (EBV), Human polyomaviruses (HPyV), and Human papillomavirus (HPV) are associated with multiple types of cancers worldwide [10, 11].

Infections caused by Epstein‐Barr (EBV) affect more than 90% of the adult population worldwide and act as an oncogenic cofactor in several lymphoid and epithelial cancers, such as Burkitt lymphoma, Hodgkin lymphoma, T‐cell lymphoma, nasopharyngeal carcinoma, gastric carcinoma and colorectal cancer [12, 13]. Interestingly, EBV and hrHPV types, especially HPV 16 and 18, are DNA viruses reported previously to be linked with 38% of all viruses associated with cancers [9, 11]. It is described that EBV can integrate into the genome of mucosal epithelia and initiate and culminate the carcinogenic process [11]. The association of EBV shedding in the female genital tract and the interaction of this virus with human epithelial malignancies prompts investigations about the role of EBV in cervical cancer [12]. Although numerous studies have proposed evidence that EBV is sexually transmitted and capable of infecting cervical cells [12, 14, 15, 16]. Moreover, some reports indicate that an increase in EBV prevalence correlates with the severity of cervical lesions [12, 16].

Viruses such as BK polyomavirus (BKPyV) and John Cunningham polyomavirus (JCPyV) are prevalent worldwide, with primary infection occurring during childhood, but can occur at age [10, 17]. These viruses establish persistent infections, remaining latent in several organs, including tonsils, lower urinary tract, lymphoid tissues, and bone marrow [17]. In immunocompetent individuals, polyomaviruses infections are asymptomatic or are associated with mild pathological changes in the respiratory and urinary tracts in immunocompetent individuals or are related to some types of cancers [10, 17]. Some studies suggest a strong association of JCPyV and anal tract cancer, especially in immunocompromised individuals, but its role remains inconclusive [18]. The association of BKPyV in anal and cervical cancer remains unclear, and in the literature this virus presents an association with urologic cancer, head and neck cancer and salivary gland disease [19]. Although some authors proposed that BKPyV can act as a co‐factor for HPV in cervical neoplasia [20].

Persistent infection with hrHPV types is a well‐established necessary factor in the development of cervical cancer [10]. However, the fact that not all women infected with hrHPV progress to malignancy suggests the involvement of additional cofactors that modulate the persistence of infection and the progression from precursor lesions to invasive cancer. Among these, EBV, BKPyV, and JCPyV have emerged as potential modulators of HPV‐associated carcinogenesis mainly in populations historically underserved and exposed to multiple vulnerabilities.

EBV, either as a coinfection or as a single infection, may enhance the oncogenic potential of hrHPV, including through mechanisms such as epithelial transformation, immune modulation, and viral synergism [5, 10, 12]. Nevertheless, the role of EBV in cervical carcinogenesis remains controversial and incompletely understood. In this context, the present study aimed to investigate the presence of EBV and other oncoviruses, BKPyV and JCPyV, in cervical and anal scrapings of women living with HIV, coinfected with HPV types.

## 2 Methods

### 2.1 Ethical Issues

Women were invited to participate in the study after signing a written informed consent form, followed by an epidemiological interview. Written informed consent was obtained from all participants in strict compliance with the Brazilian ethical guidelines involving human subjects (protocol numbers 1.099.852 and 1.059.253).

### 2.2 Study Design and Population

This is a cross‐sectional study that is part of a larger investigation conducted in the Tapajós region, Brazil, by Rodrigues and collaborators [21]. The collection of samples was carried out from August 2015 to August 2016, that included non‐indigenous women living with HIV and HPV positive in Tapajós region. The HIV‐infected women were being followed up at the Santarém counseling and testing center. This is the only referral center for the care and follow‐up of people living with HIV in this region. All women living with HIV and HPV positive were collected intravenous blood samples to prepare aliquots of serum and plasma, for conventional HIV serology confirmatory testing (4th‐generation ARCHITECT assay, Abbott, Germany) and HIV viral load determination (Abbott Real Time HIV‐1® kit, Abbott, Germany; limit of detection 40 copies/mL) and of whole blood samples for CD4 + T‐lymphocytes (Alere^TM^ PIMA CD4 Test, Jena, Germany), according to the manufacturer's instructions.

In total, 33 cervical and 30 anal scraping samples were collected, including 26 paired samples from both anatomical sites, from these women living with HIV and HPV positive at the health care provider. The cervical samples were obtained by self‐collection, and the anal scraping was performed using a brush inserted approximately 1.95 inches into the anal canal, rotated 680° five times, and subsequently preserved in *ThinPrep* Solution [21]. After the collection, biological samples were separated and sent by specialized transport to the AIDS and Molecular Immunology Laboratory at the Oswaldo Cruz Foundation and Molecular Virology and Parasitology Laboratory, Rio de Janeiro, Brazil, where the DNA extraction and viral detection process was carried out. Later, to the University of Southern California, Los Angeles, CA, USA, and to the International Agency of Research on Cancer, Lyon, France.

### 2.3 DNA Extraction

Firstly, 140 µL of samples were used for nucleic acids extraction by QIAmp DNA Mini kit (QIAGEN, Hilden, Germany), according to the manufacture's recommendations. The extracted DNA was stored at −80°C, until the time of analysis.

### 2.4 HPV Detection and Genotyping by Nested PCR, Nucleotide Sequencing and Novaplex Assay

Nested PCR was performed to detect HPV DNA targeting the L1 gene. Nucleotide sequence analysis was done using Molecular Evolutionary Genetics Analysis (MEGA) software version 7.0.18. Sequence characterization was performed in the Basic Local Alignment Search Tool using nucleotide (BLASTn) (https://blast.ncbi.nlm.nih.gov). Samples presenting DNA sequencing electropherograms with double or multiple peaks were subject to detection of multiple HPV infections using the Novaplex^TM^ II HPV28 Detection Assay (Seegene, Seoul, South Korea) in the CFX96^TM^ Real‐time PCR System (Bio‐Rad, Hercules, California, USA). The molecular assays related to diagnostic of HPV and HIV were performed in a previous study with these same women, inclued HPV samples were grouped into three groups considering the oncogenic risk classification: high‐risk, “probable” or “possible” carcinogenic and low‐risk [21, 22].

### 2.5 EBV, BKPyV and JCPyV Viral Detection and Quantification by Quantitative Real‐Time Polymerase Chain Reaction (qPCR)

And, later, at the International Agency of Research on Cancer (IARC) with the collaboration of Dr. Tarik Gheit by quantitative Real‐time (qPCR). It was performed based on using commercial TaqMan^TM^ Universal PCR Master Mix (Thermo Fischer Scientific, Waltham, MA, USA), to confirm viral detection, as well as to measure viral load through EBV, BKPyV and JCPyV target regions EBNA1 and Tag of both polyomaviruses, respectively. Multiplex qPCR was performed for JCPyV and BKPyV, while EBV was analyzed using a monoplex qPCR. The reactions were set up as follows: 12.5 µL of 2X TaqMan Universal Master Mix, 1 µL of each primer (0.4 µM), 1 µL of probe (0.2 µM), 2.5 µL of DNA, and nuclease‐free water was added to a find volume of 25 µL.

Oligonucleotides, probes and synthetic standard curves were previously described by Fellner et al. [23] and Castro et al. [24]. Synthetic standard curves ranging from 10^3^ to 10^7^ copies/mL were used for absolute viral DNA quantification. Ultrapure water and negative samples were used as negative control and positive samples were used as positive control.

### 2.6 Statistical Analysis

Descriptive statistics were performed for qualitative variables using absolute and relative frequencies. The viral loads of EBV, JCPyV, and BKPyV in anal and cervical scraping samples were described using the median and interquartile range (P25 and P75), as the data showed a nonparametric distribution. Subsequently, Student's *t*‐test was applied to assess whether coinfections with these novel oncoviruses (EBV, JCPyV, and BKPyV) were associated with HIV‐1 viral load and CD4 + T lymphocyte counts. To evaluate whether cervical or anal infection by EBV, JCPyV, or BKPyV acted as a cofactor for oncogenesis or as a protective factor in the development of cervical cancer, the chi‐square test was used. For inferential statistics, a 95% confidence interval and a 5% significance level were adopted.

## 3 Results

### 3.1 Clinical and Sociodemographic Description of Women in This Study

The study population was comprised of non‐indigenous women living with HIV and HPV positive, unvaccinated for HPV. The clinicopathological data are shown in Table 1. The range of age groups was between 32 and 42 years (36.6%) (*N* = 15), with a significant proportion between 21 and 31 years (22.0%) (*N* = 9). Reproductive history showed that 46.3% (*N* = 19) of participants had four or more pregnancies. The number of sexual partners varied widely, with 34.1% (*N* = 14) reporting ten or more lifetime partners, and condom use during vaginal intercourse was low (29.3%) (*N* = 12). A total of 36.6% (*N* = 15) reported a history of sexually transmitted infections, with genital warts being the most common condition (22.0%) (*N* = 9). Risky sexual behaviors were frequent: 53.7% (*N* = 22) reported engaging in anal intercourse, generally without condom use (29.3%) (*N* = 12), and 34.1% (*N* = 14) reported oral intercourse without condom use. Regarding cervical cancer screening, inflammatory changes were predominant (51.2%) (*N* = 21), followed by negative results for malignancy (36.6%) (*N* = 15), while low‐ and high‐grade cervical lesions were identified in 7.3% (*N* = 3) and 4.9% (*N* = 2) of women, respectively.

**Table 1:** Data of the epidemiological characteristics of women living with HIV and HPV positive in Tapajós region, Amazon, Brazil.

Variable	(%)	Total
Age		
< 20	12.2	5
21–31	22.0	9
32–42	36.6	15
43–53	17.1	7
≥ 54	12.2	5
Number of pregnancies		
None	7.3	3
1	9.8	4
2–3	29.3	12
4 or more	46.3	19
Not reported	7.3	3
Number of sexual partners		
1	7.3	3
2–4	31.7	13
5–9	24.4	10
≥ 10	34.1	14
Not reported	2.4	1
Type of STI		
Genital warts (HPV)	22.0	9
Herpes	2.4	1
Syphilis	9.8	4
Gonorrhea	2.4	1
Not reported	2.4	1
Vaginal intercourse with condom use		
No	70.7	29
Yes	29.3	12
Anal intercourse		
No	46.3	19
Yes	53.7	22
Anal intercourse with condom		
No	29.3	12
Yes	24.4	10
Not reported	46.3	19
Oral intercourse		
No	56.1	23
Yes	43.9	18
Oral intercourse with condom		
No	34.1	14
Yes	9.8	4
Not reported	56.1	23
Conclusive cervical cancer screening		
Negative for malignancy	36.6	15
Low‐grade lesion	7.3	3
High‐grade lesion	4.9	2
Inflammation	51.2	21

### 3.2 HPV Frequency and Classification in Cervical and Anal Scrapings by Nested PCR, Nucleotide Sequencing and Novaplex Assay

**Figure 1:**
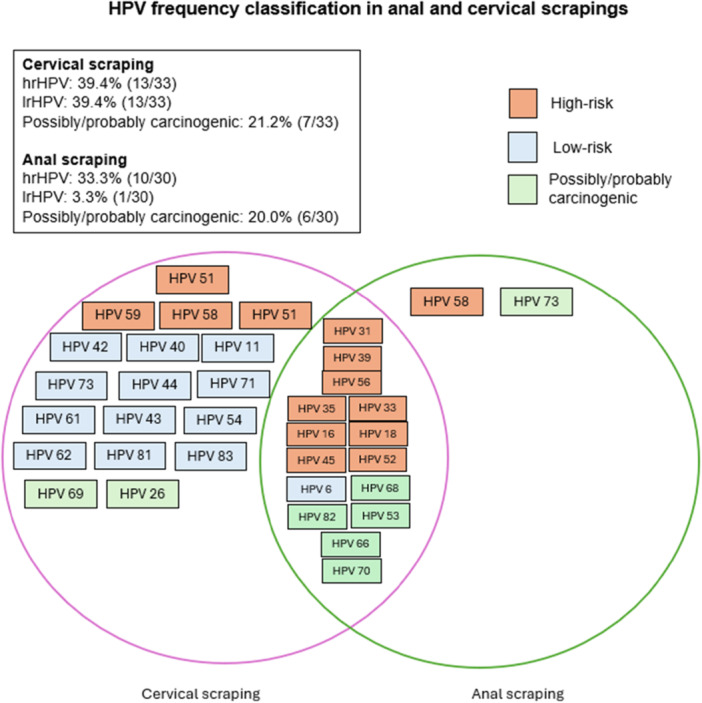
HPV frequency and classification in cervical and anal scrapings. The purple circle is related to cervical scraping and the green circle to anal scraping. The HPV were classified in three colors, hrHPV in orange, lrHPV in blue and possibly/probably carcinogenic in green.

The high‐risk (hrHPV), probably/possibly low‐risk (lrHPV) carcinogenic HPV genotypes were identified (Figure 1). A high rate of hrHPV was observed in both types of samples, 39.4% (*N* = 13/33) in cervical scrapings and 33.3% (*N* = 10/30) in anal scrapings. Although HPV 51 was more detected in cervical scrapings and HPV 56 in anal scrapings.

HPV types as HPV 31, 39, 56, 35, 33, 16, 18, 45 and, 52 as hrHPV, HPV 68, HPV82, HPV53, HPV 66 and HPV70 as possibly/probably carcinogenic and HPV 6 as lrHPV were common in both types of samples (Figure 1).

### 3.3 Frequency of Detection of EBV, JCPyV and Bkpyv in Cervical and Anal Scrapings in Women Living With HIV and HPV Positive

In relation to EBV, JCPyV and BKPyV viral detection, EBV presented a rate of 21.2% (*N* = 7) in cervical scraping and 33.3% (*N* = 10) in anal scraping, while JCPyV and BKPyV prevalence rates were equal to or lower than 10% across both types of samples (Table 2).

**Table 2:** Viral detection of EBV, JCPyV and BKPyV in cervical and anal scrapings.

	Cervical scraping *N* (%)	Anal scraping *N* (%)
**Parameters**		
*EBV*		
Positive	7 (21.2)	10 (33.3)
Negative	26 (78.8)	20 (66.7)
*JCPyV*		
Positive	4 (9.8)	3 (10.0)
Negative	29 (70.7)	27 (90.0)
*BKPyV*		
Positive	1 (2.4)	1 (3.3)
Negative	32 (78.0)	29 (96.7)

### 3.4 Viral Load of EBV, JCPyV and BKPyV in Cervical and Anal Scrapings in Women Living With HIV and HPV Positive

**Figure 2:**
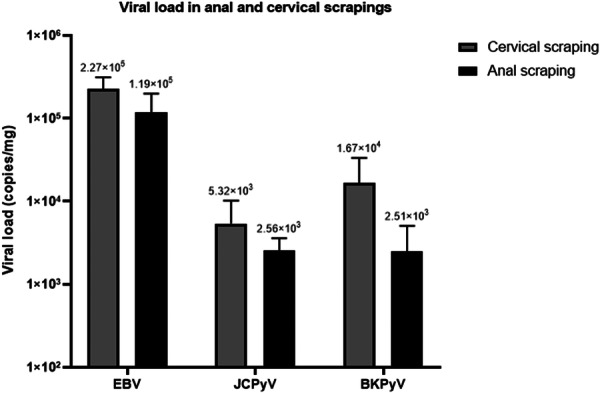
Viral load in cervical and anal scrapings. The gray bars refer to cervical scraping, and the black bars refer to anal scraping.

When analyzing the two types of samples, cervical scraping appeared to show higher viral loads than anal scraping, although no statistical comparison was performed (Figure 2). Among the viruses analyzed, EBV appeared to exhibit the highest viral load in cervical scraping (2.27 × 10^5; ±7.75 × 10^4), followed by anal scraping (1.19 × 10^5; ± 8.11 × 10^4), in contrast to JCPyV and BKPyV.

### 3.5 Coinfections of EBV, JCPyV and BKPyV With hrHPV, Possibly/Probably Carcinogenic and lrHPV Detected in Cervical and Anal Scrapings

HPV genotyping revealed both single and multiple infections in anal and cervical samples. In several cases, more than one HPV genotype was detected in the same sample, as shown in Table 3. Most samples showed no coinfection between these viruses and HPV. However, among the coinfected cases, EBV was more frequently detected in association with hrHPV genotypes in both sample types (14.6%). Regarding specific HPV genotypes, HPV 31 was the most common type coinfected with EBV, while HPV 58 was most frequently detected with JCPyV in anal scrapings. In cervical scrapings, a similar pattern was observed: HPV 31 was again the most frequent genotype in EBV‐positive samples, HPV 16 was more frequently detected in JCPyV‐positive samples.

**Table 3:** Description of EBV, JCPyV and BKPyV coinfected with high‐risk HPV, possibly/probably carcinogenic and low‐risk HPV in cervical and anal scrapings.

Patient	Age (Years)	Paired Samples	EBV (Copies/mL)	JCPyV (Copies/mL)	BKPyV (Copies/mL)	High‐risk HPV	Possible/Probably carcinogenic	Low‐risk HPV
112	51	Anal	ND	ND	ND	56	69, 82	—
112	51	Cervical	2.48 × 10^5	ND	ND	35, 45, 56, 59	—	44, 61
113	38	Anal	5.40 × 10^5	ND	ND	31, 35, 45, 56, 58	66, 68	—
113	38	Cervical	2.48 × 10^5	ND	ND	—	68	—
114	56	Anal	1.35 × 10^5	ND	ND	31	—	75
114	56	Cervical	ND	ND	ND	51, 59	82	42
115	34	Anal	ND	2.56 × 10^3	ND	58	—	—
115	34	Cervical	ND	ND	ND	51, 59	—	42
117	17	Anal	7.43 × 10^4	ND	ND	—	—	—
117	17	Cervical	ND	ND	ND	35, 52	70	61, 81
121	44	Anal	9.46 × 10^7	ND	ND	56	82	—
121	44	Cervical	1.57 × 10^6	ND	ND	31, 58	68	11, 40, 73
123	40	Anal	1.32 × 10^6	ND	ND	31, 33, 39, 56	68	—
123	40	Cervical	2.27 × 10^5	6.96 × 10^5	1.67 × 10^4	45, 51	69,82	42, 81, 103
129	30	Anal	4.26 × 10^5	ND	ND	31, 35, 58	68	121
129	30	Cervical	ND	ND	ND	31, 52, 58	68	40, 42
133	32	Anal	ND	ND	2.51 × 10^3	56	—	121
133	32	Cervical	ND	ND	ND	31, 51, 59	82	42
134	49	Anal	ND	ND	ND	35	70	—
134	49	Cervical	ND	5.42 × 10^3	ND	—	—	—
138	26	Anal	8.34 × 10^4	1.02 × 10^3	ND	—	—	—
138	26	Cervical	ND	ND	ND	—	—	62
140	18	Anal	ND	ND	ND	16	—	—
140	18	Cervical	1.08 ×10^5	4.78 × 10^3	ND	39	—	54, 81
141	46	Anal	8.76 × 10^4	6.04 × 10^3	ND	16, 18, 31, 35, 39, 52, 58	82	
141	46	Cervical	ND	ND	ND	16, 51	53	42
147	57	Anal	1.03 × 10^5	ND	ND	35, 56	—	—
147	57	Cervical	ND	ND	ND	18, 33, 35, 39, 45	69	6, 43, 54, 61
148	36	Anal	ND	ND	ND	56	—	—
148	36	Cervical	8.75 × 10^4	ND	ND	18, 51, 59	82	6, 42
149	43	Anal	2.84 × 10^5	ND	ND	56	53	—
149	43	Cervical	8.55 × 10^4	ND	ND	16, 56	53	—
153	33	Anal	ND	ND	ND	16	—	—
153	33	Cervical	ND	5.22 × 10^3	ND	45	—	42, 24, 76

Abbreviation: ND – non‐detected.

### 3.6 Association Between EBV, JCPyV and BKPyV Detection and Cytopathological Diagnoses in Cervical Scraping

The cytological profiles of cervical scrapings were analyzed according to positive and negative samples for EBV, JCPyV and BKPyV No statistically significant association was observed between the presence of viruses and cytological parameters (low‐ and high‐grade cervical lesion). In EBV, JCPyV and BKPyV, most positive cases were associated with benign or inflammatory changes.

### 3.7 Impact of EBV, JCPyV, and BKPyV Coinfections on HIV‐1 Viral Load and CD4 + T Cell Levels

No significant differences in HIV‐1 viral loads were observed between women positive or negative for EBV, JCPyV, or BKPyV in either cervical or anal scrapings (*p* > 0.05 for all comparisons) (Table 4). In cervical samples, JCPyV‐positive women showed a trend toward higher HIV‐1 viral loads (mean 3.44 ± 0.79 copies/mL) compared with JCPyV‐negative ones (2.76 ± 0.57 copies/mL), although this difference was not statistically significant (*p* = 0.112).

**Table 4:** Association between coinfections with EBV, JCPyV and, BKPyV and Immunological parameters (HIV‐1 viral load and CD4 + T cell count).

Parameters	*N*	Mean	Std. deviation	*T*‐Student
*Viral loads (copies/mL)*				
**Cervical scraping**				
EBV				0.389
*Positive*	7	1596.71	411.198	
Negative	26	632.62	2070.597	
JCPyV				
*Positive*	4	2765.00	5420.047	0.112
*Negative*	29	571.21	1965.162	
BKPyV				
*Positive*	1	87.00	—	0.773
*Negative*	32	860.56	2618.791	
**Anal scraping**				
EBV				
*Positive*	10	282.80	754.249	0.501
*Negative*	20	869.05	2643.681	
JCPyV				
*Positive*	3	39.00	0.000	0.607
*Negative*	27	744.15	2311.016	
BKPyV				
*Positive*	1	66.00	—	0.784
*Negative*	29	694.59	2234.640	
*CD4+ levels (cells/mm* ^*3*^ *)*				
**Cervical scraping**				
EBV				
*Positive*	7	320.86	149.793	0.168
*Negative*	26	477.23	280.180	
JCPyV				0.181
*Positive*	4	276.75	147.176	
*Negative*	29	467.14	269.895	
BKPyV				0.260
*Positive*	1	147.00	—	
*Negative*	32	453.34	262.802	
**Anal scraping**				
EBV				0.253
*Positive*	10	354.10	203.668	
*Negative*	20	476.45	297.464	
JCPyV				0.685
*Positive*	3	497.67	285.157	
*Negative*	27	428.78	275.882	
BKPyV				**0.039**
*Positive*	1	983.00	—	
*Negative*	29	416.79	256.656	

Regarding CD4 + T cell counts, no significant associations were found between these viruses and CD4+ levels (*p* > 0.05). Nonetheless, a tendency toward lower CD4 + T cell counts was observed among EBV‐and JCPyV‐positive women compared to negative individuals in cervical scrapings. Interestingly, BKPyV positivity in anal scrapings was associated with a higher CD4 + T cell count (983.00 vs 416.79 cells/mm^3^, *p* = 0.039).

## 4 Discussion

In this study, we showed a high EBV detection in 33.3% of anal scrapings and 21.2% of detection in cervical scraping. Commonly, people living with HIV are more susceptible to acquire multiple infections caused by some herpesviruses, including EBV, when compared to the general population [25, 26]. The EBV and HPV share similar routes and sites of infection, increasing the probability of coinfections [14, 27]. In literature, some studies demonstrated that EBV has been identified as the most prevalent herpesvirus in cervical samples from women living with HIV, as demonstrated by Cameron and collaborators in a cohort study that simultaneously assessed the presence of HPV and EBV [28]. Consistent with these findings, our study also observed a high prevalence of EBV in this population. Although traditionally associates with latent infection in B cells, EBV can also be detected in mucosal sites such as the anal and cervical tracts, particularly among individuals living with HIV and those engaging in receptive anal intercourse [29]. Despite limited reports, available evidence indicates that EBV may be present in anal samples from both HIV‐positive and HIV negative individuals, with a notably higher prevalence among those with HIV [30], as observed in our cohort.

In another aspect, EBV was found in anal and cervical scrapings coinfected with hrHPV, and HPV 31 genotype was the most frequent in both types of samples, followed by HPV 51 and HPV 56. A previous review showed that coinfections with EBV and hrHPV are associated with an increased risk of cervical abnormalities and cancer development, acting as cofactors in the carcinogenic process [31]. In this interaction, HPV is considered the primary driver, while EBV may contribute by promoting an immunosuppressive microenvironment, increasing HPV DNA integration, and altering host gene expression [29]. The carcinogenic process may involve the activity of EBV oncoproteins (LMP1, LMP2A, and EBNA1) and HPV oncoproteins (E5 and E6/E7) [29]. These oncoproteins can cooperate to initiate or amplify epithelial‐mesenchymal transition (EMT) events, one of those hallmarks of cancer progression and metastasis [29]. In a study with a cohort of 332 sex worker women demonstrated that the presence of EBV can increase the prevalence of hrHPV infection when compared to EBV‐negative women [32]. In this context, given the immunocompromised status, it is evident that women living with HIV have an increased risk of acquiring coinfections and experiencing EBV reactivation. Consequently, the likelihood of detecting hrHPV coinfection is higher, and these women are more susceptible to virus‐associated carcinogenesis. Although most studies have evaluated EBV coinfection with hrHPV in general, the biological significance of coinfection involving specific HPV‐31 remains poorly understood. Therefore, the coinfection observed in our study may suggest a potential interaction between these viruses, but further studies are needed to clarify its biological and clinical relevance.

In addition to the high frequency of EBV in the samples, an elevated viral load of this virus was also observed, particularly in cervical scrapings. The investigation of EBV viral load in cervical samples remains an area of ongoing research, with relatively few studies specifically addressing this topic. Similarly, Silver and collaborators investigated 2,331 women and detected HPV, HCMV, and EBV in vaginal scrapings [33]. Although EBV showed a high viral load in these samples, no statistically significant association was observed.

Although no statistically significant association was found in our study between the cytological profiles of the women and the presence of EBV, JCPyV, or BKPyV, it is known that infection of cervical cells by these viruses may induce the release of pro‐inflammatory cytokines and activate signaling pathways involved in inflammatory through viral persistence [34, 35]. Several studies have reported that cervical lesions frequently present lymphocytic infiltrates, which may reflect a local immune response or immunological modulation associated with viral infection [35, 36, 37]. However, this interpretation should be approached with caution, as direct evidence supporting this mechanism in cervical tissues remains limited. Therefore, these findings should be considered a hypothesis that warrants further investigation. In this context, EBV and polyomaviruses may contribute to the multifactorial process of cervical carcinogenesis, potentially by sustaining and inflammatory microenvironment in cervical tissue.

In addition to the EBV and polyomaviruses found in this study, HPV 51 and HPV 56 were most detected in cervical and anal scrapings, respectively. In a pre‐immunization survey conducted in North Sardinia, Italy, HPV 51 was identified in 19.4% of invasive cervical cancers [38]. Similarly, in large population‐based studies from China and Turkey, HPV 51 ranked among the most frequent high‐risk types, ranging from 1.5% to 10% of positive cervical samples [39, 40]. These findings suggest that HPV‐51 remains an important high‐risk genotype not covered by the current quadrivalent vaccine. Regarding to the anal region, studies conducted among transgender women in Rio de Janeiro, Brazil, found HPV‐56 in 12.5% of anal samples [41], and additional analyses among men who had sex with men (MSM) living with HIV demonstrated a significant association between HPV‐56 infection and high‐grade anal intraepithelial lesions (HSIL) [42]. These findings highlight the widespread presence of HPV 51 in the cervix and HPV 56 in the anal tract, supporting the data of this study.

Whereas our results did not show statistically significant differences in HIV‐1 viral load or CD4 + T cell counts according to coinfection status with EBV, JCPyV and BKPyV, the trends observed are consistent with prior evidence that EBV may be associated with higher HIV viral loads and altered immune status in people living with HIV. In literature, studies have found that HIV‐positive individuals with detectable EBV DNA had significantly higher HIV viral loads than those without [43, 44]. In contrast, JCPyV and BKPyV have shown weaker or no clear associations with CD4+ counts in HIV populations, suggesting their role in modulating HIV‐related immune parameters may be limited [45]. Given our sample size and the small number of positives, particularly for BKPyV, these findings should be interpreted with caution, and further longitudinal studies with larger cohorts are needed to clarify whether such coinfections contribute meaningfully to immune or virological outcomes in women living with HIV.

Although Epstein‐Barr virus (EBV) is not officially classified as a sexually transmitted infection, sexual transmission has been suggested as possible route. The virus is primarily spread through saliva; however, EBV DNA has also been detected in genital secretions, including cervical mucus and semen, indicating that intimate sexual contact may facilitate transmission. Studies have reported higher EBV detection rates in the genital tract among individuals with multiple sexual partners and in populations with coinfections such as HIV or HPV [15, 30]. Thus, although EBV is not epidemiologically categorized as an STI, sexual transmission may contribute to its transmission, particularly in high‐risk groups. In this study, 43.7% of women reported engaging in anal intercourse, and 29.3% reported practicing it without condom use. Previous studies have demonstrated the presence of EBV in semen, the male urethral tract, and the female cervical region. In a study involving heterosexual couples, identical viral isolates were detected in both partners, supporting the possibility of sexual transmission of EBV [15]. Furthermore, another investigation involving MSM and heterosexual men detected EBV DNA in 8 of 27 anal samples from MSM and in 3 of 34 samples from heterosexual men, suggesting that the anal region may serve as a reservoir for subclinical EBV infection [30]. These findings support the detection of EBV in anal samples observed in the present study.

Given the capacity of EBV, JCPyV, and BKPyV to develop cancer, this aspect emphasizes the importance of detecting these viruses in individuals with cancer, especially those living with HIV and coinfected with HPV. The detection of EBV and HPV in both the cervical and anal regions can be explained by their shared routes of transmission, the epithelial tropism of both viruses, and local immune vulnerability [46, 47, 48, 49]. The cervix and anal canal are composed of transition zones rich in basal epithelial cells, which are highly susceptible to viral entry and persistence. Sexual practices involving these sites facilitate simultaneous exposure to both viruses. Additionally, local micro abrasions, inflammation, or immunosuppression, particularly in people living with HIV, can enhance viral persistence and reactivation. HPV does not depend on EBV to cause cancer. High‐risk HPV (hrHPV) infections alone are sufficient to drive carcinogenesis through the expression of viral oncoproteins such as E6 and E7, which inactivate p53 and pRb [50]. However, EBV may act as a cofactor in the carcinogenic process [51]. EBV can promote chronic inflammation, inhibit apoptosis, and modulate host immune responses, creating a microenvironment that may favor HPV persistence and progression from infection to lesion or malignancy.

Although this study presents some limitations that should be considered when interpreting the findings. These limitations included a low number of samples size and an absence of follow‐up of these patients to observe prospective evolution of cervical lesions, as well the detection and viral load. Thus, EBV/HPV co‐infection should be interpreted as a potential synergistic interaction, rather than a dependency. Coinfection may increase the risk of malignant transformation by combining the oncogenic mechanisms of HPV with the pro‐inflammatory and immunomodulatory effects of EBV. This interplay may be particularly relevant in immunocompromised populations and in anatomical sites with shared epithelial vulnerability, such as the cervix and anal mucosa. This information reinforces the need for further investigations through the viral interactions with cancer pathways.

## Author Contributions

All attributions were equally divided among the authors, as well as the two herein carried out research activities and the preparation of the present study.

## Disclosure

Where authors are identified as personnel of the International Agency for Research on Cancer/World Health Organization, the authors alone are responsible for the views expressed in this article and they do not necessarily represent the decisions, policies, or views of the International Agency for Research on Cancer/World Health Organization.

## Ethics Statement

The current cross‐sectional and retrospective study was approved by Brazilian ethical guidelines involving human subjects (protocol numbers 1.099.852 and 1.059.253).

## Consent

Written informed consent was obtained from all individual participants included in the study.

## Conflicts of Interest

The authors declare no conflicts of interest.

## Permission to Reproduce Material From Other Sources

This manuscript does not contain any previously published material requiring permission for reproduction.

## Data Availability

The authors have nothing to report.
